# Attitudes of radiologists and interns toward the adoption of GPT-like technologies: a National Survey Study in China

**DOI:** 10.1186/s13244-025-01908-8

**Published:** 2025-01-31

**Authors:** Tianyi Xia, Shijun Zhang, Ben Zhao, Ying Lei, Zebin Xiao, Bingwei Chen, Junhao Zha, Yaoyao Yu, Zhijun Wu, Chunqiang Lu, Tianyu Tang, Yang Song, Yuancheng Wang, Shenghong Ju

**Affiliations:** 1 Nurturing Center of Jiangsu Province for State Laboratory of AI Imaging & Interventional Radiology (Southeast University), Department of Radiology, Zhongda Hospital, Medical School of Southeast University, Nanjing, China; 2 Department of Biomedical Sciences, University of Pennsylvania, Philadelphia, PA USA; 3 Department of Epidemiology and Biostatistics, School of Public Health, Southeast University, Nanjing, China; 4 MR Research Collaboration Team, Siemens Healthineers Ltd., Shanghai, China

**Keywords:** Radiologists, Attitude, Surveys and questionnaires, Natural language processing

## Abstract

**Objectives:**

To investigate the attitudes of Chinese radiologists or interns towards generative pre-trained (GPT)-like technologies.

**Methods:**

A prospective survey was distributed to 1339 Chinese radiologists or interns via an online platform from October 2023 to May 2024. The questionnaire covered respondent characteristics, opinions on using GPT-like technologies (in clinical practice, training and education, environment and regulation, and development trends), and their attitudes toward these technologies. Logistic regression was conducted to identify underlying factors associated with the attitude.

**Results:**

After quality control, 1289 respondents (median age, 37.0 years [IQR, 31.0–44.0 years]; 813 males) were surveyed. Most of the respondents (*n* = 1223, 94.9%) supported adoption of GPT-like technologies. Based on the acceptance level of GPT-like technologies, the respondents were 3 (0.2%), 29 (2.2%), 352 (27.3%), 677 (52.5%), and 228 (17.7%) from low to high acceptance degrees. Multivariable analysis revealed significant associations between positive attitudes towards GPT-like technologies and their acceptance: writing papers and language polishing (odds ratio [OR] = 1.99; *p* < 0.001), influence of colleagues using such technologies (OR = 1.77; *p* = 0.007), government regulation introduction (OR = 2.25; *p* < 0.001), and enhancement of decision support capabilities (OR = 2.67; *p* < 0.001). Sensitivity analyses confirmed these results for different acceptance thresholds (all *p* < 0.001).

**Conclusions:**

Chinese radiologists or interns generally support GPT-like technologies due to their potential capabilities in clinical practice, medical education, and scientific research. They also emphasize the need for regulatory oversight and remain optimistic about their future medical applications.

**Critical relevance statement:**

This study highlights the broad support among Chinese radiologists for GPT-like technologies, emphasizing their potential to enhance clinical decision-making, streamline medical education, and improve research efficiency, while underscoring the need for regulatory oversight.

**Key Points:**

- The impact of GPT-like technologies on the radiology field is unclear.
- Most Chinese radiologists express the supportive adoption of GPT-like technologies.
- GPT-like technologies’ capabilities at research and clinic prompt the attitude.

**Graphical Abstract:**

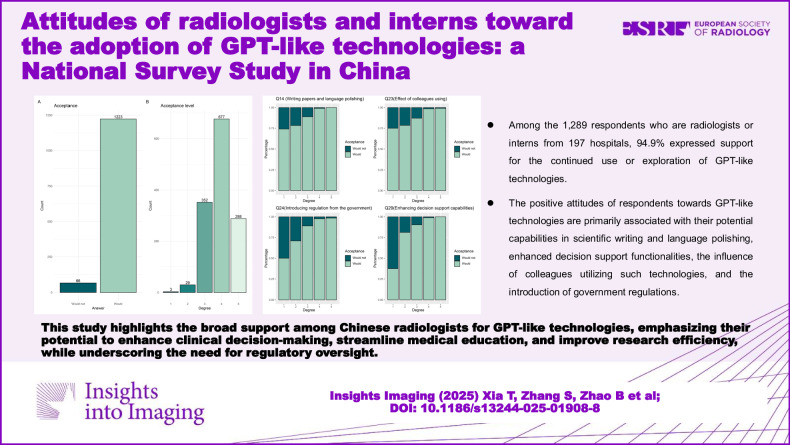

## Introduction

The series of ChatGPT (e.g., GPT-3.5 and GPT-4) and other tools based on large language models (LLMs) have initiated the latest wave of chatbots, impacting numerous industries. These powerful chatbots, designed to interpret, generate, and effectively converse with humans, are referred to as GPT (generative pre-trained)-like technologies, such as ChatGPT, Claude, and Google Bard. The development and refining of LLMs have also significantly impacted the field of healthcare [1]. The potential applications of GPT-like technologies in healthcare have garnered significant attention, particularly in areas such as scientific writing and medical education [2–4].

Healthcare is inundated with large amounts of free-text reports, such as radiological reports, which place a significant burden on radiologists. The inconsistencies in style and structure result in variability and complexity in radiological reports, hindering effective communication of information among various medical departments [5]. GPT-like technologies have shown great potential in structuring and correcting errors in free-text radiological reports and providing therapeutic suggestions through these reports [6–9]. Recently, positive conclusions regarding the detection of radiological findings in chest radiographs using GPT-4 with vision capabilities have demonstrated its immense potential in the department of radiology [10, 11]. Therefore, GPT-like technologies have the potential to support clinical workflow, scientific research, and educational applications in the field of radiology [3].

However, ChatGPT-like technologies have not been universally successful across all domains. Some studies indicate that, while GPT-4 demonstrated moderate agreement with human-assigned breast nodule categories, it still produced a high percentage of discordant results, potentially impacting clinical decision-making [12]. Furthermore, while GPT-like models excelled in text-based tasks, issues such as repeatability, demographic biases, and challenges in interpreting radiologic images persisted [13–15]. These limitations underscored critical areas for improvement, highlighting the need for further development before clinical implementation.

The rapid growth of interest in GPT-like technologies in healthcare raised several challenges, particularly regarding ethical concerns and potential risks building on this controversial evidence, such as misdiagnosis or missed diagnoses [2, 16, 17]. Currently, the acceptance and understanding of GPT-like technologies among radiologists are still unclear and need further investigation, which may influence the future deployment of these technologies in the field of radiology. The Zhongda Radiology Alliance, established in 2019, is a collaborative network of radiology academic centers. As of the time of our survey, it includes 264 hospitals across 31 of China’s 34 provinces. Conducting this survey within the framework of the Zhongda Radiology Alliance ensures a highly representative sample of the Chinese radiology community.

Therefore, our study aimed to conduct a comprehensive national survey to elucidate the attitudes and possible influencing factors of Chinese radiologists or interns regarding the use of GPT-like technologies in their clinical practice, medical education, and scientific research.

## Materials and methods

This cross-sectional study was approved by the research ethics board and informed consent was acquired from all participants. The study was conducted in accordance with both the Declarations of Helsinki and Istanbul. This study adheres to the Strengthening the Reporting of Observational Studies in Epidemiology (STROBE) reporting guideline.

### Survey time and procedure

The survey was conducted from October 2023 to May 2024. Questionnaires were distributed to members of the Zhongda Radiology Alliance via the survey platform *Wenjuanxing* (https://www.wjx.cn/) in China. The questionnaires were forwarded to radiologists or interns willing to participate through the directors of the department of radiology. Standard instructions for the questionnaire survey were provided, and appointed people were assigned to be responsible for the distribution, and quality control of the questionnaires. The questionnaires were anonymous, and all items were mandatory to complete.

### Questionnaire formulation and survey respondents

The questionnaire was designed after repeated discussions and revisions by hospital management experts, senior radiology experts, and artificial intelligence (AI) algorithm experts (the questionnaire was detailed in Supplemental Materials). The questionnaire comprised three parts. Part-A: basic information included gender, age, province of workplace, hospital level, working history, professional title, scientific research pressure, experience with GPT-like technologies, familiarity with AI, and familiarity with GPT-like technologies. Part B: options on GPT-like technologies, in which the respondents rated their opinion on a Likert scale from 1 (very poor) to 5 (very good) concerning GPT-like technologies in clinical practice (10 items), training and education (9 items), environment and regulation (6 items), and development trends (5 items). Besides, Part C consisted of 2 key questions: 1. Without considering policies and costs, would you be willing to try or continue using GPT-like technologies? (would not; would); and 2. Would you be willing to try or continue using GPT-like technologies? (definitely would not; probably would not; neutral; probably would; definitely would).

**Fig. 1:**
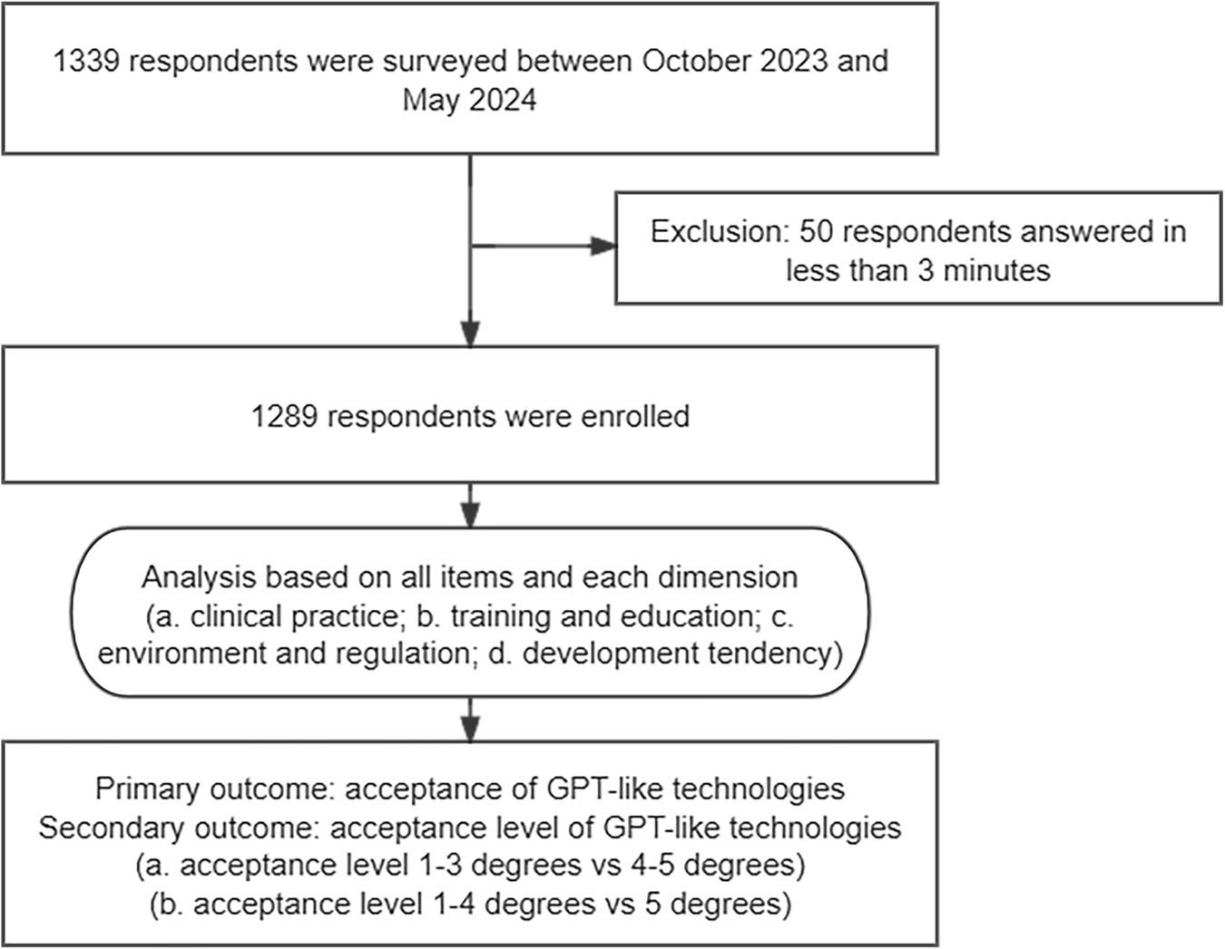
The process and analysis of the survey study

All participants completed the survey anonymously without the collection of any identifiers. The respondents were radiologists with registered physician qualifications or interns majoring in radiology in mainland China. A total of 1340 respondents were members of the Zhongda Radiology Alliance from 197 hospitals. For quality control, we empirically excluded the 50 respondents who completed the questionnaire in less than 3 min (Fig. 1). The primary outcome of this study was attitudes toward the adoption of GPT-like technologies, and the secondary outcome was to explore the factors that affect the attitudes.

### Statistical analysis

Results conforming to a normal distribution were expressed as mean and standard deviation, while categorical data were presented as frequencies and percentages. Results conforming to a non-normal distribution and ordered categorical data were expressed as the median and interquartile range (IQR). The Mann–Whitney *U*-test, or Chi-square test was performed based on the variable type. Correlations between ordered categorical variables were assessed using Spearman correlation analysis. Univariable and multivariable logistic regression was conducted to identify independent factors, presented with odds ratios (OR) and 95% confidence interval (CI). Variables with a *p* value < 0.1 and potential bias in the univariate logistic regression analysis were included in the multivariable analysis. The multivariable logistic regression analysis was applied to identify factors associated with radiologists’ attitudes toward the acceptance of GPT-like technologies, using the minimum value of the Akaike Information Criterion. Additionally, stratified analysis was performed as a sensitivity analysis to control the effect of confounders with potential bias.

For sensitivity analysis, we re-divided the acceptance level of GPT-like technologies into two groups: 1–3 degrees vs 4–5 degrees, and 1–4 degrees vs 5 degrees. Multivariable logistic regression analysis was performed as described above for overall items and each of the four dimensions: clinical practice, training and education, environment and regulation, and development tendency. All *p* values were two-sided, and significance was set at *p* value < 0.05. Survey data were analyzed using SPSS statistical software version 27.0 (IBM).

## Results

### Characteristics of the respondents

**Fig. 2:**
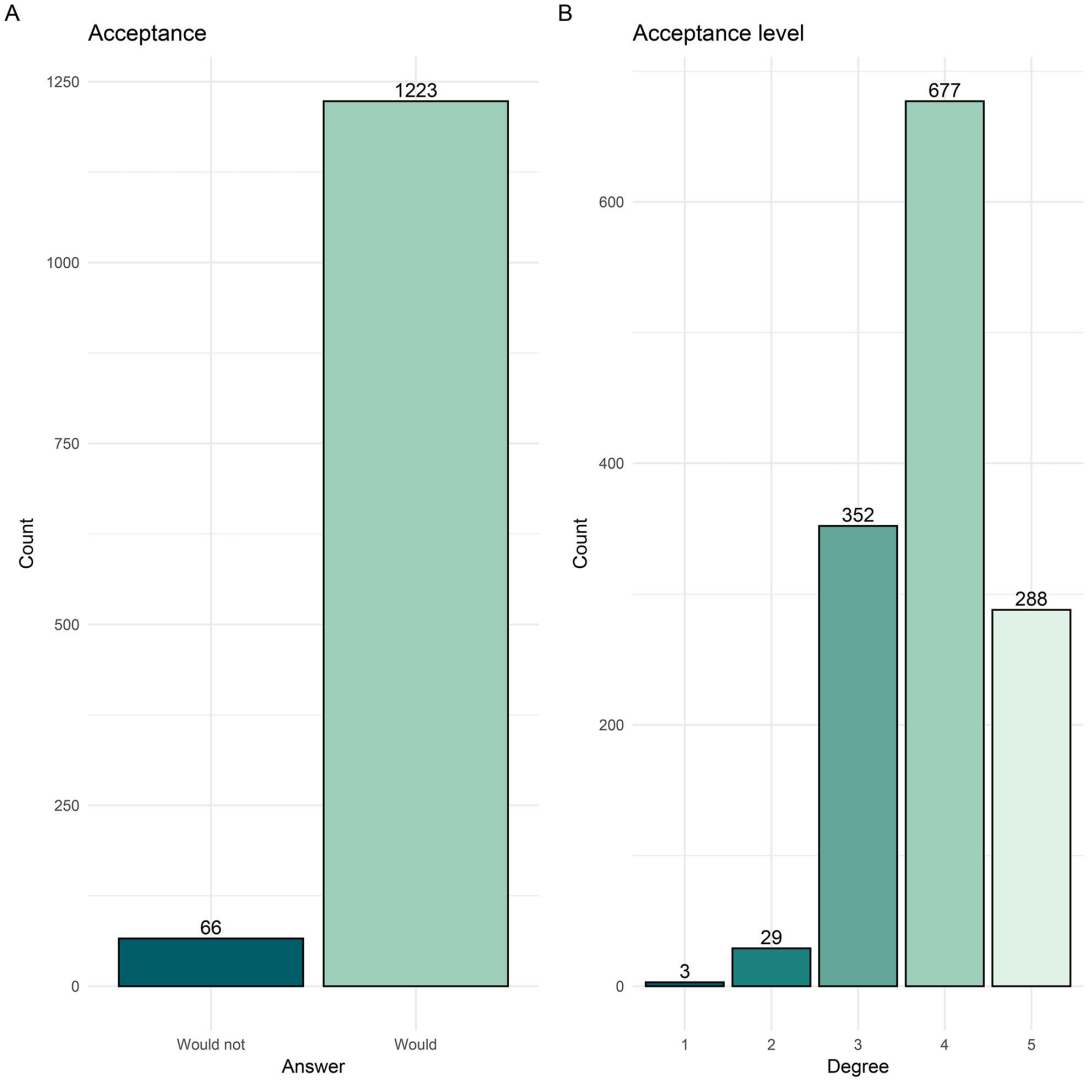
Bar graphs depicting respondents’ attitudes toward the adoption of GPT-like technologies based on two key questions (Supplemental Materials). **A** represents attitudes categorized into two categories, while **B** represents attitudes based on a 5-point ordinal scale. GPT, generative pre-trained

The study ultimately enrolled 1289 respondents after excluding 50 respondents due to quality control. All respondents completed the survey, and the response rate reached 96.3% without 50 unqualified questionnaires. The respondents were from 197 hospitals in 27 provinces, with a median age of 37.0 years (IQR, 31.0–44.0 years) and a median working history of 12 years (IQR, 7–21 years). Among them, 813 were males. A majority of respondents (1066, 82.7%) were from tertiary hospitals. The professional titles were distributed as follows: 438 (34.0%) were associate chief or chief radiologists, 508 (39.4%) were attending radiologists, and 343 (26.6%) were interns or residents. Notably, 125 respondents (9.7%) reported prior experience with GPT-like technologies. Respondents experiencing greater scientific research pressure (*p* = 0.03), having a longer working history (*p* = 0.04), or holding higher professional titles (*p* = 0.02) were more likely to try and continue using GPT-like technologies. One thousand two hundred twenty-three (94.9%) respondents expressed support for GPT-like technologies (Fig. 2A). Based on the acceptance level, the respondents were distributed as 3 (0.2%), 29 (2.2%), 352 (27.3%), 677 (52.5%), and 228 (17.7%) from low to high acceptance degrees of GPT-like technologies (Fig. 2B). Detailed characteristics of the respondents are provided in Table 1.

**Table 1:** Characteristics of the respondents (*n* = 1289)

Characteristics	Respondents (*n* = 1289)	Opponents (*n* = 66)	Supporters (*n* = 1223)	*p* value
Sex				0.53
Female	476 (36.9)	22 (33.3)	454 (37.1)	
Male	813 (63.1)	44 (66.7)	769 (62.9)	
Age (years)^*^	37.0 (31.0–44.0)	35.0 (29.8–40.0)	37.0 (31.0–44.0)	0.051^†^
Region				0.42
Northeast	59 (4.6)	3 (4.5)	56 (4.6)	
North	136 (10.6)	9 (13.6)	127 (10.4)	
East	459 (35.6)	15 (22.7)	444 (36.3)	
Southern	30 (2.3)	1 (1.5)	29 (2.4)	
Central	158 (12.3)	11 (16.7)	147 (12)	
Northwest	58 (4.5)	3 (4.5)	55 (4.5)	
Southwest	389 (30.2)	24 (36.4)	365 (29.8)	
Hospital level				0.13
Tertiary	1066 (82.7)	50 (75.8)	1016 (83.1)	
Others	223 (17.3)	16 (24.2)	207 (16.9)	
Working history (years)^*^	12.0 (7.0–21.0)	10.0 (6.0–17.0)	12.0 (7.0–21.0)	0.04^†^
Professional title				0.02^†^
Intern or resident	343 (26.6)	23 (34.8)	320 (26.2)	
Attending	508 (39.4)	29 (43.9)	479 (39.2)	
Associate chief or chief	438 (34.0)	14 (21.2)	424 (34.7)	
Academic pressure				0.24^†^
None	269 (20.9)	21 (31.8)	248 (20.3)	
Mild	249 (19.3)	7 (10.6)	242 (19.8)	
Moderate	367 (28.5)	21 (31.8)	346 (28.3)	
Moderately severe	186 (14.4)	6 (9.1)	180 (14.7)	
Severe	218 (16.9)	11 (16.7)	207 (16.9)	
Used experience with GPT-like technologies				0.86
Yes	125 (9.7)	6 (9.1)	119 (9.7)	
No	1164 (90.3)	60 (90.9)	1104 (90.3)	
Knowledge of NLP or AI technologies				0.27^†^
No understanding	110 (8.5)	9 (13.6)	101 (8.3)	
Limited understanding	291 (22.6)	16 (24.2)	275 (22.5)	
Basic understanding	425 (33.0)	19 (28.8)	406 (33.2)	
Good understanding	456 (35.4)	22 (33.3)	434 (35.5)	
Excellent understanding	7 (0.5)	0 (0.0)	7 (0.6)	
Knowledge of GPT-like technologies				0.29^†^
No understanding	218 (16.9)	16 (24.2)	202 (16.5)	
Limited understanding	556 (43.1)	24 (36.4)	532 (43.5)	
Basic understanding	427 (33.1)	25 (37.9)	402 (32.9)	
Good understanding	62 (4.8)	0 (0.0)	62 (5.1)	
Excellent understanding	26 (2.0)	1 (1.5)	25 (2.0)	
Acceptance level of GPT-like technologies				< 0.001^†^
Definitely would not	3 (0.2)	3 (4.5)	0 (0.0)	
Probably would not	29 (2.2)	8 (12.1)	21 (1.7)	
Neutral	352 (27.3)	45 (68.2)	307 (25.1)	
Probably would	677 (52.5)	10 (15.2)	667 (54.5)	
Definitely would	228 (17.7)	0 (0.0)	228 (18.6)	

Unless otherwise specified, data are numbers of patients, with percentages in parentheses

*AI* artificial intelligence, *GPT* generative pre-trained, *NLP* natural language processing

^*^ Data are medians, with IQRs in parentheses

^†^ *p* value calculated by Mann–Whitney *U*-test

### Attitudes of the respondents toward the acceptance of GPT-like technologies

**Fig. 3:**
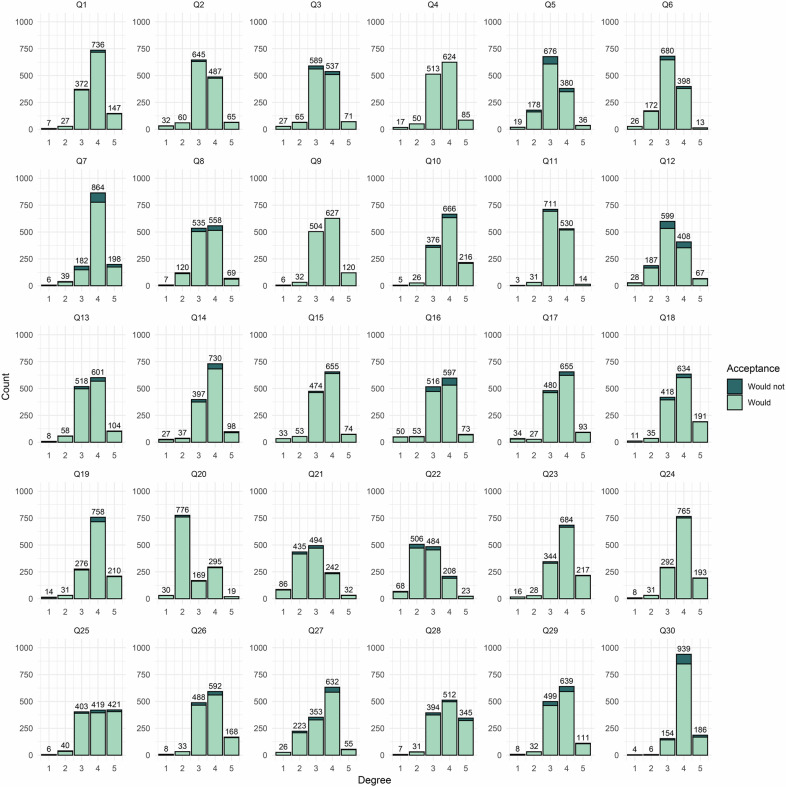
The acceptance of GPT-like technologies distribution of respondents’ answers to questions 1–30 from Part B of the questionnaire. Bar graphs show a positive trend where the majority of respondents chose a positive or neutral response (3–5 degrees) at most items. Details of the questions can be found in Supplemental Materials. GPT, generative pre-trained

The distribution of responses to 30 questions (Q1–Q30) from Part B of the questionnaire, which pertains to the attitudes of respondents towards GPT-like technologies, is presented in Fig. 3. The majority of items showed a clear trend where most respondents chose either a positive response (4–5 degrees) or a neutral response (3 degrees), indicating overall optimistic opinions regarding the potential capabilities of GPT-like technologies. Differences in attitudes towards GPT-like technologies between the opponents and supporters were observed in most items from Part B (Supplementary Table 1). Additionally, correlation analysis showed positive correlations between a majority of items and attitudes toward GPT-like technologies (Supplementary Table 2).

Univariable binary logistic regression analysis comprehensively assessed the possible factors associated with radiologists’ acceptance of GPT-like technologies. Our findings revealed that a longer working history was significantly associated with higher acceptance rates (OR = 1.03; 95% CI: 1.00, 1.06; *p* = 0.03), indicating that more experienced radiologists may be more willing to adopt GPT-like technologies. Furthermore, the univariable analysis showed that respondents’ positive attitudes towards GPT-like technologies were influenced by optimistic opinions in the four dimensions of clinical practice, training and education, environment and regulation, and development tendency (Table 2).

**Table 2:** Binary logistic regression analysis of variables for the association with acceptance of GPT-like technologies (*n* = 1289)

Factors^*^	Univariable analysis		Multivariable analysis	
	OR (95% CI)	*p* value	OR (95% CI)	*p* value
Age (years)	1.03 (0.99, 1.06)	0.06		
Working history (years)	1.03 (1.00, 1.06)	0.03		
Q1 (Assisting in writing radiological reports)	1.78 (1.27, 2.47)	< 0.001		
Q2 (Improving doctor-patient communication)	1.35 (0.99, 1.83)	0.06		
Q3 (Improving patient triage and treatment)	1.61 (1.19, 2.17)	0.002		
Q4 (Impact on the radiology department)	1.70 (1.24, 2.32)	< 0.001		
Q5 (Using in emergencies)	1.65 (1.19, 2.27)	0.003		
Q6 (Producing misleading diagnostic results)	1.04 (0.74, 1.47)	0.82		
Q7 (Role in medical image diagnosis)	1.67 (1.22, 2.30)	0.002		
Q8 (Providing personalized treatment advice)	1.58 (1.15, 2.17)	0.005		
Q9 (Reducing the radiological reporting writing time)	1.67 (1.18, 2.34)	0.004		
Q10 (Improving efficiency in radiology emergency shifts)	2.16 (1.56, 2.99)	< 0.001		
Q11 (Accurate medical information)	1.87 (1.21, 2.89)	0.005		
Q12 (Diagnostic advice in rare or complex cases)	1.35 (1.01, 1.81)	0.045		
Q13 (Improving radiology education effectiveness)	1.72 (1.23, 2.38)	0.001		
Q14 (Writing papers and language polishing)	3.36 (2.56, 4.41)	< 0.001	1.99 (1.34, 2.95)	< 0.001
Q15 (Designing research topics)	2.62 (2.01, 3.42)	< 0.001		
Q16 (Designing patents)	2.30 (1.79, 2.96)	< 0.001		
Q17 (Editing research and statistics code)	2.65 (2.03, 3.46)	< 0.001		
Q18 (Enhancing disease understanding)	3.99 (2.87, 5.54)	< 0.001		
Q19 (GPT-like tech training in medical education)	2.69 (2.04, 3.56)	< 0.001		
Q20 (Leading to medical disputes)	0.90 (0.69, 1.17)	0.43		
Q21 (Threat to radiologists’ jobs)	1.11 (0.85, 1.46)	0.44		
Q22 (Privacy leak of patients)	1.03 (0.77, 1.37)	0.87		
Q23 (Effect of colleagues using)	3.30 (2.47, 4.41)	< 0.001	1.77 (1.17, 2.68)	0.007
Q24 (Introducing regulation from the government)	3.65 (2.66, 5.02)	< 0.001	2.25 (1.56, 3.22)	< 0.001
Q25 (Supervising in medicine)	1.79 (1.34, 2.35)	< 0.001		
Q26 (Widely used in hospitals in the future)	1.74 (1.26, 2.41)	< 0.001		
Q27 (Impact on doctors’ employment)	1.19 (0.91, 1.56)	0.20		
Q28 (Application in the medicine in next decade)	1.56 (1.17, 2.08)	0.003		
Q29 (Enhancing decision support capabilities)	5.01 (3.40, 7.36)	< 0.001	2.67 (1.58, 4.49)	< 0.001
Q30 (Application in the medical industry)	3.95 (2.67, 5.84)	< 0.001		

Unless otherwise specified, Data in parentheses are 95% CIs. The multivariable analysis included variables with a *p* value < 0.1 and potential bias (used experience with GPT-like technologies, knowledge of NLP/AI technologies and GPT-like technologies)

*AI* artificial intelligence, *CI* confidence interval, *GPT* generative pre-trained, *NLP* natural language processing, *OR* odds ratio

^*^ Summary of the item are shown in parentheses, the details of the items can be found in the Supplementary File

**Fig. 4:**
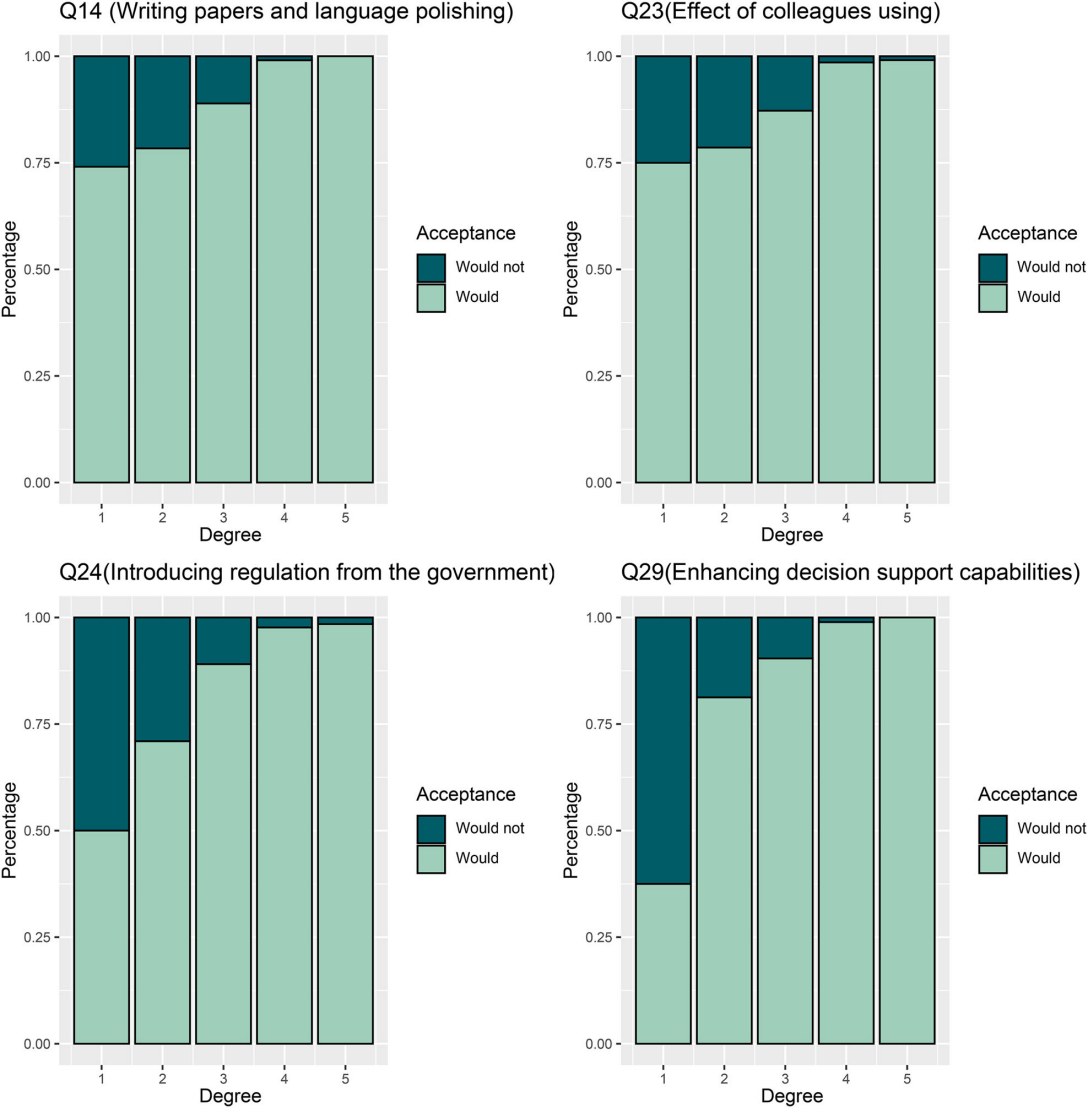
Bar graphs show the cumulative percentage of respondents’ answers to questions 14, 23, 24, and 29. As the response level increases to these questions, the support tendency for GPT-like technologies increases (*p* < 0.001). Details of questions 14, 23, 24, and 29 can be found in Supplemental Materials. GPT, generative pre-trained

After adjusting other factors with *p* < 0.1, the multivariable binary logistic regression analysis identified several key questions associated with the acceptance of GPT-like technologies: Q14 (Writing papers and language polishing) (OR = 1.99; 95% CI: 1.34, 2.95; *p* < 0.001), Q23 (Effect of colleagues using) (OR = 1.77; 95% CI: 1.17, 2.68; *p* < 0.007), Q24 (Introducing regulation from the government) (OR = 2.25; 95% CI: 1.56, 3.22; *p* < 0.001), and Q29 (Enhancing decision support capabilities) (OR = 2.67; 95% CI: 1.58, 4.49; *p* < 0.001). Figure 4 displays a histogram comparing responses to these four questions between supporters and opponents of GPT-like technologies.

**Fig. 5:**
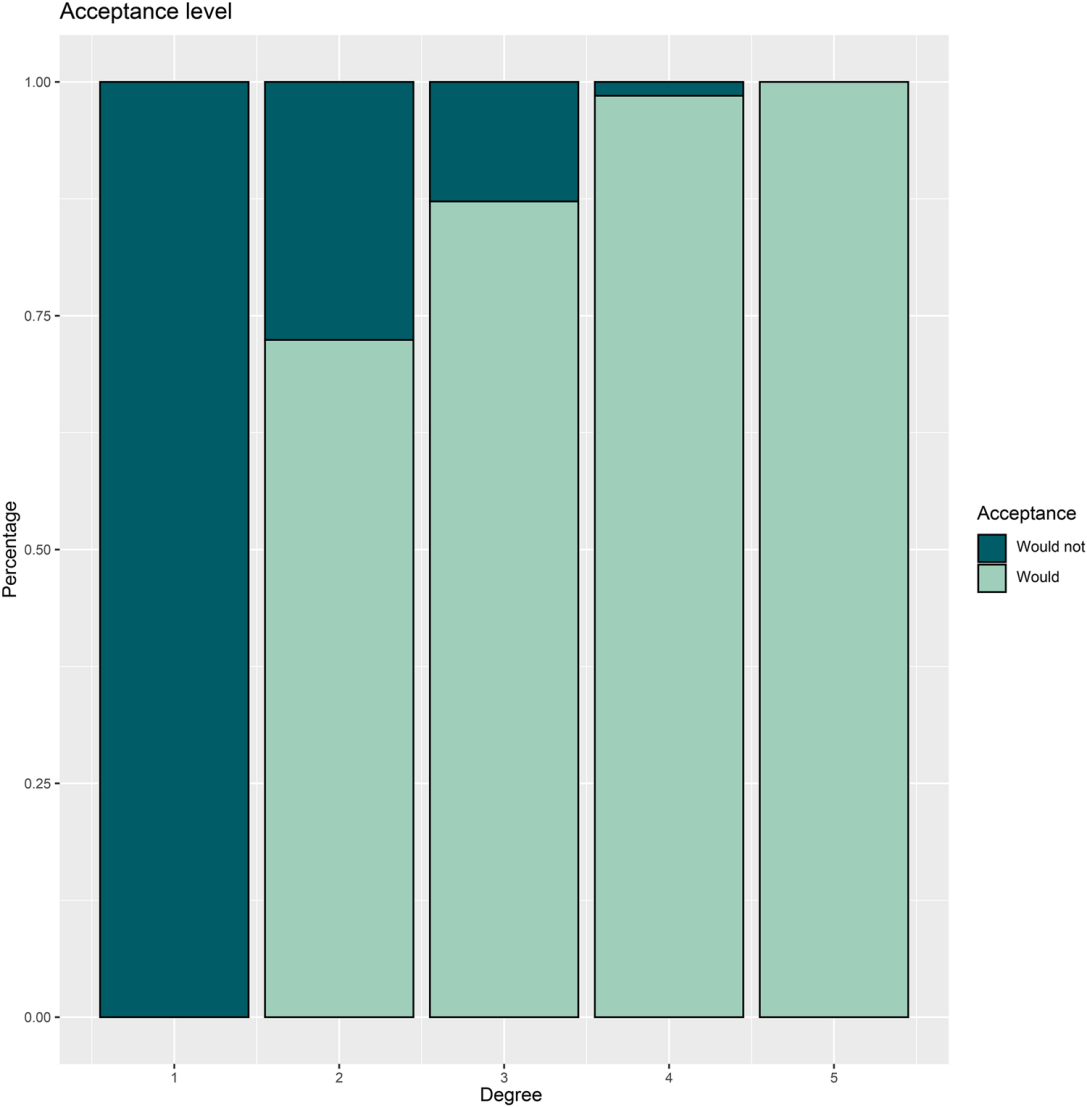
Bar graphs show the cumulative percentage between the key questions about the acceptance and acceptance level of GPT-like technologies (*p* < 0.001). GPT, generative pre-trained

To more accurately describe the acceptance of GPT-like technologies, we included two questions with 2 and 5 categories for acceptance level. The relationship between these two key questions is shown in Table 1 and Fig. 5 (*p* < 0.001). Sensitivity analyses were conducted to gain deeper insights into the factors associated with acceptance level. Regardless of whether the acceptance was categorized as 1–3 degrees vs 4–5 degrees or 1–4 degrees vs 5 degrees, the four items (Q14, Q23, Q24, and Q29) were still the independent factors associated with higher acceptance levels in multivariable analysis (all *p* < 0.001) (Table 3). Given the experience of use experience of GPT-like technologies and the level of knowledge about AI and GPT-like technologies, there may be potential bias. In the subgroup analysis, all four items (Q14, Q23, Q24, and Q29) demonstrated a significant positive association across all groups, as detailed in Table 4.

**Table 3:** Binary logistic regression analysis of variables for the association with an acceptance level of GPT-like technologies at questions 14, 23, 24, and 29 (*n* = 1289)

Factors^*^	Acceptance level (1–3 degrees vs 4–5 degrees)				Acceptance level (1–4 degrees vs 5 degrees)			
	Univariable analysis		Multivariable analysis		Univariable analysis		Multivariable analysis	
	OR (95% CI)	*p* value	OR (95% CI)	*p* value	OR (95% CI)	*p* value	OR (95% CI)	*p* value
Q14	5.07 (4.05, 6.35)	< 0.001	1.84 (1.41, 2.41)	< 0.001	9.27 (6.51, 13.21)	< 0.001	2.02 (1.35, 3.03)	< 0.001
Q23	17.00 (12.59, 22.97)	< 0.001	8.47 (6.17, 11.61)	< 0.001	25.50 (17.58, 36.99)	< 0.001	12.50 (8.30, 18.84)	< 0.001
Q24	3.94 (3.18, 4.87)	< 0.001	2.11 (1.61, 2.78)	< 0.001	5.64 (4.25, 7.47)	< 0.001	1.92 (1.33, 2.77)	< 0.001
Q29	6.21 (4.86, 7.94)	< 0.001	2.50 (1.84, 3.41)	< 0.001	7.75 (5.70, 10.53)	< 0.001	2.11 (1.45, 3.06)	< 0.001

Unless otherwise specified, Data in parentheses are 95% CIs

*CI* confidence interval, *GPT* generative pre-trained, *OR* odds ratio

^*^ The details of questions 14, 23, 24, and 29 can be found in the Supplementary File

**Table 4:** Binary logistic regression analysis of variables for the association with acceptance of GPT-like technologies at questions 14, 23, 24, and 29 in different subgroups (*n* = 1289)

Subgroup	Q14 OR (95% CI)	Q23 OR (95% CI)	Q24 OR (95% CI)	Q29 OR (95% CI)
Used experience with GPT-like technologies				
Yes	3.36 (1.28, 8.82)	2.63 (1.07, 6.49)	3.61 (1.35, 9.66)	6.61 (1.65, 26.55)
No	3.36 (2.53, 4.47)	3.38 (2.49, 4.60)	3.66 (2.62, 5.12)	4.88 (3.27, 7.29)
Knowledge of NLP or AI technologies				
No to limited understanding	3.60 (2.25, 5.77)	3.15 (1.95, 5.09)	3.01 (1.80, 5.04)	4.39 (2.34, 8.14)
Basic to excellent understanding	3.23 (2.30, 4.52)	3.37 (2.34, 4.85)	4.05 (2.70, 6.07)	5.37 (3.28, 8.81)
Knowledge of GPT-like technologies				
No to limited understanding	3.62 (2.50, 5.24)	3.28 (2.28, 4.72)	2.94 (1.89, 4.56)	4.92 (2.98, 8.12)
Basic to excellent understanding	3.07 (2.04, 4.61)	3.36 (2.07, 5.44)	4.68 (2.90, 7.57)	5.14 (2.81, 9.43)

Unless otherwise specified, Data in parentheses are 95% CIs

*AI* artificial intelligence, *CI* confidence interval, *GPT* generative pre-trained, *NLP* natural language processing, *OR* odds ratio

### The main factors affecting the attitude toward the acceptance of GPT-like technologies in each dimension

To further elucidate the multi-faceted views of radiologists’ attitudes toward GPT-like technologies, we conducted a multivariable logistic regression analysis to identify factors significantly associated with acceptance and different levels of acceptance across four dimensions. Table 5 shows the independent factors affecting the acceptance of GPT-like technologies in each dimension.

**Table 5:** Binary logistic regression analysis of variables for the association with acceptance of GPT-like technologies based on each dimension (*n* = 1289)

Factors^*^	Acceptance (no vs yes)		Acceptance level (1–3 degrees vs 4–5 degrees)		Acceptance level (1–4 degrees vs 5 degrees)	
	OR (95% CI)	*p* value	OR (95% CI)	*p* value	OR (95% CI)	*p* value
Clinical practice						
Q7 (Role in medical image diagnosis)			1.27 (1.03, 1.56)	0.02		
Q9 (Reducing the radiology reporting writing time)					1.49 (1.14, 1.95)	0.003
Q10 (Improving efficiency in radiology emergency shifts)	2.16 (1.56, 2.99)	< 0.001	1.74 (1.43, 2.11)	< 0.001	1.45 (1.12, 1.88)	0.005
Training and education						
Q14 (Writing papers and language polishing)	2.39 (1.71, 3.32)	< 0.001			2.94 (1.93, 4.94)	< 0.001
Q15 (Designing research topics)			1.53 (1.13, 2.07)	0.005		
Q16 (Designing patents)			1.86 (1.37, 2.53)	< 0.001		
Q18 (Enhancing disease understanding)	2.36 (1.56, 3.57)	< 0.001	7.67 (5.58, 10.56)	< 0.001	5.90 (3.92, 8.90)	< 0.001
Q19 (GPT-like tech training in medical education)	1.50 (1.05, 2.14)	0.03	2.02 (1.54, 2.64)	< 0.001	3.65 (2.41, 5.54)	< 0.001
Environment and regulation						
Q23 (Effect of colleagues using)	2.66 (1.91, 3.70)	< 0.001	12.84 (9.42, 17.50)	< 0.001	19.19 (13.06, 28.18)	< 0.001
Q24 (Introducing regulation from the government)	2.61 (1.85, 3.68)	< 0.001	1.99 (1.51, 2.62)	< 0.001	2.41 (1.71, 3.41)	< 0.001
Q25 (Supervising in medicine)			1.50 (1.23, 1.84)	< 0.001		
Development tendency						
Q28 (Application in the medicine in next decade)			1.38 (1.16, 1.65)	< 0.001	1.46 (1.17, 1.82)	< 0.001
Q29 (Enhancing radiologists’ decision support capabilities)	4.45 (2.94, 6.75)	< 0.001	4.21 (3.28, 5.42)	< 0.001	4.47 (3.21, 6.23)	< 0.001
Q30 (Application in the medical industry)	2.53 (1.69, 3.77)	< 0.001	5.48 (3.81, 7.89)	< 0.001	6.83 (4.66, 10.03)	< 0.001

Unless otherwise specified, Data in parentheses are 95% CIs

*CI* confidence interval, *GPT* generative pre-trained, *OR* odds ratio

^*^ Summary of the item are shown in parentheses, the details of the items can be found in the Supplementary File

In the dimension of clinical practice, radiologists’ opinions on the role of GPT-like technologies in medical image diagnosis (OR = 1.27; *p* = 0.02) and their ability to reduce radiology report writing time (OR = 1.49; *p* = 0.003) were associated with higher levels of acceptance of GPT-like technologies. Notably, positive opinions on improving efficiency in radiology emergency shifts demonstrated the strongest association with the acceptance of GPT-like technologies (all *p* < 0.05).

Regarding training and education, enhancing disease understanding of GPT-like technologies (OR = 2.36; *p* < 0.001) and incorporating GPT-like technologies training in medical education (OR = 1.50; *p* = 0.03) were strongly related to acceptance. Besides, the capability of GPT-like technologies in writing papers and language polishing, designing research topics, and designing patents were also potential factors contributing to positive attitudes toward GPT-like technologies (all *p* < 0.05).

For environment and regulation, the influence of colleagues using GPT-like technologies (OR = 2.66; *p* < 0.001) and the introduction of government regulation for GPT-like technologies (OR = 2.61; *p* < 0.001) were the most significant factors for a positive attitude. Supervising in healthcare also showed significance in the analysis of acceptance level (1–3 degrees vs 4–5 degrees) (OR = 1.50; *p* < 0.001).

Concerning the dimension of development tendency, positive opinions on the enhancement of decision support capabilities by GPT-like technologies and their application in the medical industry were the most influential factors for respondents’ positive attitude towards trying or continuing to use the GPT-like technologies (all *p* < 0.001).

## Discussion

GPT-like technologies have experienced rapid growth and garnered significant attention in radiology [1, 2, 4]. The advent of GPT has infused new energy into the field of radiology, but it has also raised new concerns, making radiologists’ opinions on these technologies crucial. Our study conducted a national survey of Chinese radiologists and interns at the Zhongda Radiology Alliance with a 96.3% response rate, revealing that the majority of the 1223 respondents (94.9% of 1289) from 197 hospitals in 27 provinces expressed support for trying or continuing to use GPT-like technologies. Furthermore, we identified several factors significantly associated with the acceptance of GPT-like technologies, including writing papers and language polishing, the effect of colleagues using such technologies, introducing regulation from the government, and enhancing decision support capabilities (all *p* < 0.001).

The Zhongda Radiology Alliance offered a unique advantage for this survey, as it spans a wide geographic area and includes a diverse range of hospitals across China. The alliance provided a high representation of the radiology community. This diversity ensured that the findings reflected the perspectives of radiologists from various settings, making the results more generalizable. The increasing demand for imaging examinations has outpaced the availability of skilled radiologists, intensifying the workload and increasing the likelihood of errors in radiology [18]. Previous studies have shown that integrating GPT-like technologies into clinical practice has the potential to structure and detect errors in radiological reports with a high cost-benefit ratio [8, 9]. In the clinical practice dimension, respondents who believed GPT-like technologies could enhance medical image diagnosis and reduce report writing time were more likely to accept them (*p* = 0.02–0.003). Additionally, their potential to improve efficiency during radiology emergency shifts was crucial to acceptance (all *p* < 0.05). Another study reported that GPT-4 achieved an accuracy of 0.88 in assessing clinical acuity in the department of emergency, compared to human accuracy of 0.86 [19]. The strong association between the belief in improved decision support (all *p* < 0.001) and the acceptance of GPT-like technologies underscores their critical role in clinical judgment and decision-making. Analyses of LLMs have concluded that these models can provide appropriate imaging examination suggestions and aid in surgical decision-making [6, 7, 20].

The integration of GPT-like technologies into medical education and scientific research presents a transformative opportunity for radiologists [2, 4]. In a survey study, over two-thirds of 263 medical students agreed on the need for AI to be included in medical training [21]. Focusing on the education dimension, our study demonstrated a positive association with the high acceptance level of GPT-like technologies, particularly in writing papers and language polishing, designing research topics and patents, and enhancing disease understanding (*p* < 0.05). Recently, the use of tools based on LLMs for polishing scientific work has become acceptable, though it raises dilemmas regarding originality and inexpressiveness [1]. These tools have also been used to assess the risk of bias in randomized clinical trials, achieving high correct assessment rates [22]. However, recent studies have also revealed limitations of GPT-like technologies in terms of general performance in radiological knowledge [23]. Indeed, respondents expressed significant concern about the accuracy of medical information provided by these technologies (*p* = 0.005 in univariable analysis). Training in GPT-like technologies within medical education was identified as an important factor associated with a positive attitude (all *p* < 0.03). However, the performance of ChatGPT-like technologies at direct imaging interpretation may not be as accurate as expected [12–15]. This could pose risks to both education and clinical practice, potentially increasing the likelihood of errors and negatively impacting patient care.

Additionally, GPT-like technologies introduce a set of challenges that must be addressed to ensure their responsible and effective use, such as medical malpractice liability, privacy, and others [16, 17]. Legal regulation is a key challenge for LLMs. Our results show that government regulations were strongly associated with tool acceptance (all *p* < 0.001), highlighting the need for developer-regulator collaboration. Additionally, the influence of colleagues using GPT-like technologies was a major factor in fostering a positive attitude (all *p* < 0.001). Future regulations for LLMs remain an unmet need and should aim to strike a balance between promoting efficiency and mitigating potential risks, with a focus on flexible, forward-looking frameworks that safeguard both societal and individual interests [24]. In our study, respondents expressed a moderately optimistic opinion about the future medical application of GPT-like technologies. A 2021 survey revealed that only 48% (501 of 1041) of radiologists had a positive attitude toward AI, which contrasts with our findings in China [25]. This discrepancy may arise from differences in survey populations or the focus on GPT-like technologies in our study. Additionally, recent AI integration in radiology may have reduced workload, enhancing radiologists’ confidence.

There were several limitations in this study. First, it is a cross-sectional study, and the participants were members of the Zhongda Radiology Alliance, which may not be representative of radiologists worldwide. None of the respondents were engaged in ultrasound diagnosis. Second, only 125 (9.7%) of the 1289 respondents had prior experience with GPT-like technologies, and most respondents may have had an inaccurate understanding of these technologies due to a lack of direct experience. Third, the survey was conducted over seven months and did not focus on a specific GPT-like tool. Thus, it is important to interpret the results with caution.

## Conclusions

Our study highlights that most radiologists or interns in mainland China display a favorable attitude toward incorporating GPT-like technologies into their clinical practice and research. This favorable attitude is driven by the popularity and superior capabilities of GPT-like technologies for scientific writing and decision support. Additionally, the establishment of sound regulations emerges as a pivotal factor in the application of GPT-like technologies in radiology.

## Supplementary information

ELECTRONIC SUPPLEMENTARY MATERIAL

## Data Availability

The related data can be obtained by contacting the corresponding author upon reasonable request.

## Abbreviations

AI: Artificial intelligence
GPT: Generative pre-trained
LLM: Large language model
