# Supplementary material for: Attitudes of radiologists and interns toward the adoption of GPT-like technologies: a National Survey Study in China
Source: Insights Imaging. 2025 Jan 31;16:30. doi: 10.1186/s13244-025-01908-8 (PMC11785863; doi:10.1186/s13244-025-01908-8)
Supplement: Supplementary file 1 — ELECTRONIC SUPPLEMENTARY MATERIAL [file 13244_2025_1908_MOESM1_ESM.docx]

**Supplemental file**

1. **Text**

**Questionnaire**

***A. Basic Information***

1. Your Gender? [Single Choice]

○ Female

○ Male

2. Your Age (in years)? [Fill in the Blank]

3. The Province You Are Located In? [Fill in the Blank]

4. What is the Level of Your Hospital? [Single Choice]

○ Tertiary General Hospital

○ Tertiary Specialized Hospital

○ Secondary General Hospital

○ Secondary Specialized Hospital

○ Other

5. How Many Years of Experience Do You Have in Radiology? [Fill in the Blank]

6. What is Your Professional Title?

○ Intern

○ Resident Doctor

○ Attending Physician

○ Associate Chief Physician

○ Chief Physician

7. Do you currently experience academic pressure? [Single Choice]

○ None

○ Mild

○ Moderate

○ Moderately severe

○ Severe

8. Have you used GPT-like Natural Language Processing tools before? [Single Choice]

○ No

○ Yes

9. Are you familiar with Natural Language Processing (NLP) or other Artificial Intelligence (AI) technologies? [Single Choice]

○ No understanding

○ Limited Understanding

○ Basic Understanding

○ Good Understanding

○ Excellent Understanding

10. How well do you understand GPT-like technologies? [Single Choice]

○ No understanding

○ Limited Understanding

○ Basic Understanding

○ Good Understanding

○ Excellent Understanding

***B. Questionnaire Question*** (Options are rated on a Likert scale from 1 [very poor] to 5 [very good])

***B-1. Clinical Practice***

**Q1.** What is your opinion on the potential effectiveness of GPT-like technologies in assisting with the writing of radiology reports? [Single Choice]

**Q2.** Do you believe GPT-like technologies can improve doctor-patient communication and patient understanding of radiology reports? [Single Choice]

**Q3.** Do you believe GPT-like technologies can improve patient triage and treatment? [Single Choice]

**Q4.** How much impact do you think GPT-like technologies can have in healthcare, especially in your department? [Single Choice]

**Q5.** What is your opinion on the potential of GPT-like technologies in emergency situations (such as providing advice in case of contrast agent allergy)? [Single Choice]

**Q6.** Are you concerned about the potential for GPT-like technologies to produce misleading diagnostic results? [Single Choice]

**Q7.** Do you believe that GPT-like technologies can play a role in medical image diagnosis? [Single Choice]

**Q8.** Do you believe that GPT-like technologies can provide personalized treatment recommendations and advice? [Single Choice]

**Q9.** What is your opinion on the potential for GPT-like technologies to reduce the time required for radiology report writing? [Single Choice]

**Q10.** Do you think GPT-like technologies can improve efficiency in radiology emergency shift? [Single Choice]

***B-2. Training and Education***

**Q11.** Do you think the medical information provided by GPT-like technologies is accurate enough? [Single Choice]

**Q12.** Do you think GPT-like technologies can provide diagnostic advice for rare cases or complex situations? [Single Choice]

**Q13.** Do you think GPT-like technologies can help improve the effectiveness of radiology education and training? [Single Choice]

**Q14.** Would you consider using GPT-like technologies to assist you in writing papers, including language polishing? [Single Choice]

**Q15.** Would you consider using GPT-like technologies to assist you in designing research topics? [Single Choice]

**Q16.** Would you consider using GPT-like technologies to assist you in designing patents? [Single Choice]

**Q17.** Would you consider using GPT-like technologies to assist you in editing code for research and statistics? [Single Choice]

**Q18.** Would you use GPT-like technologies to enhance your understanding of diseases? [Single Choice]

**Q19.** Do you think medical education should include training and education on GPT-like technologies? [Single Choice]

***B-3. Environment and Regulation***

**Q20.** Are you concerned that the application of GPT-like technologies might lead to medical disputes? [Single Choice]

**Q21.** Are you concerned that GPT or similar technologies might replace some radiologists’ jobs? [Single Choice]

**Q22.** Are you concerned about privacy protection issues that GPT-like technologies might raise? [Single Choice]

**Q23.** Would you start using GPT-like technologies because colleagues and friends around you are using them? [Single Choice]

**Q24.** Do you think the government should introduce laws and regulations related to the medical applications of GPT-like technologies? [Single Choice]

**Q25.** Do you think the application of GPT-like technologies in the medical industry should be supervised? [Single Choice]

***B-4. Development Tendency***

**Q26.** Do you think it is likely that GPT-like technologies will be widely used in hospitals? [Single Choice]

**Q27.** Do you think the use of GPT-like technologies will affect doctors’ employment? [Single Choice]

**Q28.** Do you think GPT-like technologies might be applied in the Chinese medical industry in the next decade? [Single Choice]

**Q29.** Do you believe GPT-like technologies can enhance radiologists’ decision support capabilities? [Single Choice]

**Q30.** What is your attitude towards the future application of GPT-like technologies in the medical industry? [Single Choice]

***C. Key Question***

1. Without considering policies and costs, would you be willing to try or continue using GPT-like technologies? [Single Choice] *

○ Would not

○ Would

2. Would you be willing to try or continue using GPT-like technologies? [Single Choice] *

○ Definitely would not

○ Probably would not

○ Neutral

○ Probably would

○ Definitely would

***A summary of items of Part B***

1. Assisting in writing radiology reports
2. Improving doctor-patient communication
3. Improving patient triage and treatment
4. Impact on the Radiology Department
5. Using in emergencies
6. Producing misleading diagnostic results
7. Role in medical image diagnosis
8. Providing personalized treatment advice
9. Reducing the radiology reporting writing time
10. Improving efficiency in radiology emergency shifts
11. Accurate medical information
12. Diagnostic advice in rare or complex cases
13. Improving radiology education effectiveness
14. Writing papers and language polishing
15. Designing research topics
16. Designing patents
17. Editing research and statistics code
18. Enhancing disease understanding
19. GPT-like technology training in medical education
20. Leading to medical disputes
21. Threat to radiologists’ jobs
22. Privacy leak of patients
23. Effect of colleagues using
24. Introducing regulation from the government
25. Supervising in medicine
26. Widely used in hospitals in the future
27. Impact on doctors’ employment
28. Application in the medicine in next decade
29. Enhancing radiologists’ decision support capabilities
30. Application in the medical industry
31. **Table**

**Table S1** Questions of the Respondents (*n* = 1,289)

| Factors | Respondents  (*n* = 1289) | Opponents  (*n* = 66) | Supporters  (*n* = 1223) | *p* value |
| --- | --- | --- | --- | --- |
| Q1. What is your opinion on the potential effectiveness of GPT-like technologies in assisting with the writing of radiology reports? | 4 (3-4) | 3.5 (3-4) | 4 (3-4) | .003 |
| Q2. Do you believe GPT-like technologies can improve doctor-patient communication and patient understanding of radiology reports? | 3 (3-4) | 3 (3-4) | 3 (3-4) | .06 |
| Q3. Do you believe GPT-like technologies can improve patient triage and treatment? | 3 (3-4) | 3 (3-4) | 3 (3-4) | .01 |
| Q4. How much impact do you think GPT-like technologies can have in healthcare, especially in your department? | 4 (3-4) | 3 (3-4) | 4 (3-4) | .002 |
| Q5. What is your opinion on the potential of GPT-like technologies in emergency situations (such as providing advice in case of contrast agent allergy)? | 3 (3-4) | 3 (3-3) | 3 (3-4) | .003 |
| Q6. Are you concerned about the potential for GPT-like technologies to produce misleading diagnostic results? | 3 (3-4) | 3 (3-3) | 3 (3-4) | .59 |
| Q7. Do you believe that GPT-like technologies can play a role in medical image diagnosis? | 4 (4-4) | 4 (3-4) | 4 (4-4) | .005 |
| Q8. Do you believe that GPT-like technologies can provide personalized treatment recommendations and advice? | 3 (3-4) | 3 (3-4) | 3 (3-4) | .006 |
| Q9. What is your opinion on the potential for GPT-like technologies to reduce the time required for radiology report writing? | 4 (3-4) | 3 (3-4) | 4 (3-4) | .003 |
| Q10. Do you think GPT-like technologies can improve efficiency in radiology emergency shifts? | 4 (3-4) | 3 (3-4) | 4 (3-4) | < .001 |
| Q11. Do you think the medical information provided by GPT-like technologies is accurate enough? | 3 (3-4) | 3 (3-4) | 3 (3-4) | .008 |
| Q12. Do you think GPT-like technologies can provide diagnostic advice for rare cases or complex situations? | 3 (3-4) | 3 (3-3.25) | 3 (3-4) | .08 |
| Q13. Do you think GPT-like technologies can help improve the effectiveness of radiology education and training? | 4 (3-4) | 3 (3-4) | 4 (3-4) | .001 |
| Q14. Would you consider using GPT-like technologies to assist you in writing papers, including language polishing? | 4 (3-4) | 3 (3-3) | 4 (3-4) | < .001 |
| Q15. Would you consider using GPT-like technologies to assist you in designing research topics? | 4 (3-4) | 3 (3-3) | 4 (3-4) | < .001 |
| Q16. Would you consider using GPT-like technologies to assist you in designing patents? | 4 (3-4) | 3 (3-3) | 4 (3-4) | < .001 |
| Q17. Would you consider using GPT-like technologies to assist you in editing code for research and statistics? | 4 (3-4) | 3 (3-3) | 4 (3-4) | < .001 |
| Q18. Would you use GPT-like technologies to enhance your understanding of diseases? | 4 (3-4) | 3 (3-3) | 4 (3-4) | < .001 |
| Q19. Do you think medical education should include training and education on GPT-like technologies? | 4 (4-4) | 3 (3-4) | 4 (4-4) | < .001 |
| Q20. Are you concerned that the application of GPT-like technologies might lead to medical disputes? | 2 (2-3) | 2 (2-3) | 2 (2-3) | .25 |
| Q21. Are you concerned that GPT or similar technologies might replace some radiologists’ jobs? | 3 (2-3) | 3 (2-3) | 3 (2-3) | .43 |
| Q22. Are you concerned about privacy protection issues that GPT-like technologies might raise? | 3 (2-3) | 3 (2-3) | 3 (2-3) | .96 |
| Q23. Would you start using GPT-like technologies because colleagues and friends around you are using them? | 4 (3-4) | 3 (3-3) | 4 (3-4) | < .001 |
| Q24. Do you think the government should introduce laws and regulations related to the medical applications of GPT-like technologies? | 4 (3-4) | 3 (3-4) | 4 (4-4) | < .001 |
| Q25. Do you think the application of GPT-like technologies in the medical industry should be supervised? | 4 (3-5) | 3 (3-5) | 4 (3-5) | < .001 |
| Q26. Do you think it is likely that GPT-like technologies will be widely used in hospitals? | 4 (3-4) | 3 (3-4) | 4 (3-4) | .006 |
| Q27. Do you think the use of GPT-like technologies will affect doctors’ employment? | 4 (3-4) | 3 (3-4) | 4 (3-4) | .10 |
| Q28. Do you think GPT-like technologies might be applied in the Chinese medical industry in the next decade? | 4 (3-5) | 3.5 (3-4) | 4 (3-5) | .002 |
| Q29. Do you believe GPT-like technologies can enhance radiologists’ decision support capabilities? | 4 (3-4) | 3 (3-3) | 4 (3-4) | < .001 |
| Q30. What is your attitude towards the future application of GPT-like technologies in the medical industry? | 4 (4-4) | 4 (3-4) | 4 (4-4) | < .001 |

Unless otherwise specified, data are medians, with IQRs in parentheses. *GPT* Generative Pre-Trained.

**Table S2** The Correlation between Questions and Acceptance level of GPT-like Technologies (*n* =1,289)

| Question | Correlation coefficients (95% CI)* | *p* value† | Correlation coefficients (95% CI)‡ | *p* value§ |
| --- | --- | --- | --- | --- |
| Q1. What is your opinion on the potential effectiveness of GPT-like technologies in assisting with the writing of radiology reports? | 0.171 (0.115-0.225) | < .001 | 0.082 (0.026-0.138) | .003 |
| Q2. Do you believe GPT-like technologies can improve doctor-patient communication and patient understanding of radiology reports? | 0.134 (0.079-0.189) | < .001 | 0.053 (-0.003-0.109) | .057 |
| Q3. Do you believe GPT-like technologies can improve patient triage and treatment? | 0.132 (0.076-0.187) | < .001 | 0.072 (0.016-0.128) | .01 |
| Q4. How much impact do you think GPT-like technologies can have in healthcare, especially in your department? | 0.142 (0.086-0.196) | < .001 | 0.087 (0.031-0.142) | .002 |
| Q5. What is your opinion on the potential of GPT-like technologies in emergency situations (such as providing advice in case of contrast agent allergy)? | 0.143 (0.087-0.197) | < .001 | 0.082 (0.026-0.138) | .003 |
| Q6. Are you concerned about the potential for GPT-like technologies to produce misleading diagnostic results? | 0.006 (-0.050-0.063) | .82 | 0.015 (-0.041-0.071) | .59 |
| Q7. Do you believe that GPT-like technologies can play a role in medical image diagnosis? | 0.204 (0.149-0.257) | < .001 | 0.079 (0.023-0.134) | .005 |
| Q8. Do you believe that GPT-like technologies can provide personalized treatment recommendations and advice? | 0.169 (0.114-0.223) | < .001 | 0.077 (0.021-0.133) | .006 |
| Q9. What is your opinion on the potential for GPT-like technologies to reduce the time required for radiology report writing? | 0.202 (0.148-0.255) | < .001 | 0.084 (0.028-0.139) | .003 |
| Q10. Do you think GPT-like technologies can improve efficiency in radiology emergency shifts? | 0.247 (0.193-0.299) | < .001 | 0.128 (0.072-0.183) | < .001 |
| Q11. Do you think the medical information provided by GPT-like technologies is accurate enough? | 0.174 (0.119-0.228) | < .001 | 0.074 (0.017-0.129) | .008 |
| Q12. Do you think GPT-like technologies can provide diagnostic advice for rare cases or complex situations? | 0.094 (0.038-0.150) | .001 | 0.050 (-0.007-0.106) | .08 |
| Q13. Do you think GPT-like technologies can help improve the effectiveness of radiology education and training? | 0.204 (0.150-0.258) | < .001 | 0.089 (0.033-0.144) | .001 |
| Q14. Would you consider using GPT-like technologies to assist you in writing papers, including language polishing? | 0.587 (0.549-0.623) | < .001 | 0.264 (0.210-0.315) | < .001 |
| Q15. Would you consider using GPT-like technologies to assist you in designing research topics? | 0.558 (0.518-0.595) | < .001 | 0.222 (0.168-0.275) | < .001 |
| Q16. Would you consider using GPT-like technologies to assist you in designing patents? | 0.525 (0.484-0.565) | < .001 | 0.209 (0.154-0.262) | < .001 |
| Q17. Would you consider using GPT-like technologies to assist you in editing code for research and statistics? | 0.580 (0.542-0.617) | < .001 | 0.226 (0.172-0.279) | < .001 |
| Q18. Would you use GPT-like technologies to enhance your understanding of diseases? | 0.726 (0.698-0.751) | < .001 | 0.244 (0.190-0.296) | < .001 |
| Q19. Do you think medical education should include training and education on GPT-like technologies? | 0.593 (0.555-0.628) | < .001 | 0.198 (0.144-0.252) | < .001 |
| Q20. Are you concerned that the application of GPT-like technologies might lead to medical disputes? | 0.015 (-0.041-0.071) | .58 | -0.032 (-0.088-0.024) | .25 |
| Q21. Are you concerned that GPT or similar technologies might replace some radiologists’ jobs? | 0.043 (-0.013-0.099) | .12 | 0.022 (-0.034-0.078) | .43 |
| Q22. Are you concerned about privacy protection issues that GPT-like technologies might raise? | 0.025 (-0.013-0.099) | .38 | -0.002 (-0.058-0.055) | .96 |
| Q23. Would you start using GPT-like technologies because colleagues and friends around you are using them? | 0.757 (0.732-0.780) | < .001 | 0.241 (0.187-0.293) | < .001 |
| Q24. Do you think the government should introduce laws and regulations related to the medical applications of GPT-like technologies? | 0.467 (0.422-0.510) | < .001 | 0.214 (0.160-0.267) | < .001 |
| Q25. Do you think the application of GPT-like technologies in the medical industry should be supervised? | 0.365 (0.315-0.412) | < .001 | 0.110 (0.054-0.165) | < .001 |
| Q26. Do you think it is likely that GPT-like technologies will be widely used in hospitals? | 0.197 (0.143-0.251) | < .001 | 0.076 (0.020-0.132) | .006 |
| Q27. Do you think the use of GPT-like technologies will affect doctors’ employment? | 0.087 (0.031-0.143) | .002 | 0.046 (-0.010-0.102) | .10 |
| Q28. Do you think GPT-like technologies might be applied in the Chinese medical industry in the next decade? | 0.243 (0.190-0.296) | < .001 | 0.087 (0.031-0.142) | .002 |
| Q29. Do you believe GPT-like technologies can enhance radiologists’ decision support capabilities? | 0.565 (0.526-0.602) | < .001 | 0.235 (0.181-0.287) | < .001 |
| Q30. What is your attitude towards the future application of GPT-like technologies in the medical industry? | 0.519 (0.476-0.559) | < .001 | 0.197 (0.142-0.250) | < .001 |

*CI* confidence interval, *GPT* Generative Pre-Trained.

* Correlation coefficients calculated from items and acceptance of GPT-like technologies.

† *P* value calculated from items and acceptance of GPT-like technologies.

‡ Correlation coefficients calculated from items and acceptance level of GPT-like technologies.

§ *P* value calculated from items and acceptance level of GPT-like technologies.
